# Faculty development through simulation-based education in physical therapist education

**DOI:** 10.1186/s41077-017-0060-3

**Published:** 2018-01-12

**Authors:** Kristin Curry Greenwood, Sara B. Ewell

**Affiliations:** 1 Department of Physical Therapy, Movement and Rehabilitation Sciences, Northeastern University, 360 Huntington Avenue, 301M RB, Boston, MA 02115 USA; 2 College of Professional Studies, Northeastern University, Boston, MA 02115 USA

**Keywords:** Simulation-based education, Faculty development, Physical therapist educator

## Abstract

**Background:**

The use of simulation-based education (SBE) in health professions, such as physical therapy, requires faculty to expand their teaching practice and development. The impact of this teaching on the individual faculty member, and how their teaching process changes or develops, is not fully understood. The purpose of this study was to explore individual physical therapist faculty members’ experience with SBE and how those experiences may have transformed their teaching practice to answer the research questions: How do physical therapist faculty develop through including SBE and are there commonalities among educators?

**Methods:**

An interpretive phenomenological analysis approach was used with a small sample of subjects who participated in three individual semi-structured interviews. Interview questions were created through the lens of transformative learning theory to allow faculty transformations to be uncovered. A two-step thematic coding process was conducted across participants to identify commonalities of faculty experiences with SBE in physical therapist education. Credibility and trustworthiness were achieved through member checking and expert external review. Thematic findings were validated with transcript excerpts and research field notes.

**Results:**

Eight physical therapist faculty members (25% male) with a range of 3 to 16 years of incorporating SBE shared their individual experiences. Four common themes related to faculty development were identified across the participants. Themes identified are the following: faculty strengthen their professional identity as physical therapists, faculty are affected by their introduction and training with simulation, faculty develop their interprofessional education through SBE, and faculty experiences with SBE facilitate professional growth.

**Conclusion:**

Physical therapist educators had similarities in their experiences with SBE that transformed their teaching practice and professional development. This study provides insight into what physical therapist faculty may experience when adopting SBE.

## Background

Simulation-based education (SBE) is a teaching methodology that creates a learning environment where students perform psychomotor and clinical reasoning skills in a realistic and controlled environment [1]. Faculty have incorporated SBE to prepare health care providers for centuries [2]. In the late 1800s, its use in surgical practice was noted, and nursing followed in the early 1900s [2]. Faculty select from among a variety of media, ranging from simple anatomical models, to computerized manikins, to human patient actors, so the simulation creates a realistic learning experience that allows students to demonstrate skill acquisition [3]. Following the simulation performance, faculty facilitate debriefing sessions during which they guide students through reflection on their thoughts and actions to help the student attain new learning [4].

Physical therapists are part of the interprofessional medical team whose focus is to assist patients in maximizing their ability to function. In clinical practice, physical therapists utilize a combination of narrative reasoning, which is understanding and engaging with a patient’s experience, and diagnostic reasoning, which determines diagnosis, impairments, and treatment focus [5]. Simulation assists with fostering clinical development and clinical decision-making in physical therapy. Prominent modalities for SBE in physical therapist (PT) education are manikins, standardized patients, or simulated patient actors. Pritchard et al. illustrated that there is value in incorporating simulated patients in physical therapist education to enhance learning; however, studies pertaining to physical therapist education lack rigor [6]. Manikin-based SBE has also been documented as beneficial to physical therapist student learning [7, 8]. SBE has been considered as a replacement for clinical education hours in physical therapist education, as it has in other professions, such as nursing. Research has suggested that 25% of clinical education hours in physical therapist education could be replaced by SBE [6, 9].

SBE is included in multiple areas of curriculum within physical therapist education. In hospital-based physical therapy delivery, SBE assists education related to electrocardiogram interpretation, decision-making, and overall acute care clinical performance for PT students [10, 11]. In preparation for cardiorespiratory physical therapy, the integrated simulation and technology enhanced learning (ISTEL) framework has been developed, which illustrates that preparation, intervention, evaluation, and research are important components of faculty practice of SBE in physical therapist education [12]. SBE fosters the development of communication and professionalism in physical therapist preparation [6]. SBE as part of musculoskeletal teaching has increased the value of student learning when laboratory preparation time is limited [13]. The incorporation of SBE within several curricular areas emphasizes why physical therapist faculty have adopted it in their teaching practice.

Adoption of simulation-based education in any health profession, including physical therapy, requires faculty to expand their teaching practice and development [14]. SBE requires faculty to be competent in creating and assessing educational objectives for the learner and facilitating debriefing sessions where the learner reflects on their simulation performance to inform their practice [15, 16]. Faculty development in simulation, the process whereby faculty deepen their breadth and depth of expertise, benefits from structure and standards [17]. While there are standards of practice that span professions regarding development with SBE, the physical therapist faculty members’ experience of developing their skills related to SBE is not well understood. This study explores the self-reported experiences of individual physical therapist faculty who incorporate SBE in their classes to understand how these experiences may have transformed their teaching practice.

## Methods

An interpretive phenomenological analysis (IPA) approach was used to answer the research question: How do physical therapist faculty develop using SBE and are there commonalities among educators [18, 19]? Following the IPA methodology, a researcher with prior SBE experience and training conducted a series of three interviews with each participant [20]. IPA favors a small sample size in order to elicit multiple deep conversations with each individual participant, rather than single conversations with a larger cohort of participants [19]. The Seidman protocol, which uses structured follow-up interviews to obtain subjective information, was followed to provide credibility to this qualitative study [21]. This study defined SBE as including standardized or simulated patients, manikin-based simulation, or both.

Mezirow’s theory of transformative learning guided this study [22]. Transformative learning theory argues that learning in a meaningful context transforms an adult learner into a new reality by moving through stages of learning [23]. Transformative learning theory is used in SBE to guide faculty as they design and implement SBE for their students [24]. Previous studies using transformative learning theory have identified a need for further research that examines the faculty’s understanding of how SBE assists students with transformation and how participating in simulation deepens a teacher’s practice with respect to their area of expertise [25, 26]. Transformative learning theory provided a shared context for SBE and guided the semi-structured interview questions and analysis.

### Recruitment

Following the IPA methodology, participants were purposively sampled from a homogenous group within which the research question was known to have significance [27]. Therefore, a convenience sample of known PT SBE educators was identified through the primary researcher’s SBE contacts, professional business and association meetings, and authors of SBE publications and SBE sessions and/or research activity presentations. To further saturate the sample, snowballing was utilized through the first round of participants contacted. In total, 31 SBE educators were contacted. Nine accepted the invitation to participate; however, one did not respond to the next steps that would have begun the interview process. The final participant sample included eight participants, which met the IPA recommendation of six to eight participants, allowing the researcher to focus on depth not breadth of participant experiences [28].

Only physical therapist educators in the USA were included, in order to align teaching standards and curriculum for all participants. Prior to study acceptance, participants were identified as having a common use and understanding of the principles of SBE through a preliminary screening questionnaire (Appendix 1). SBE was defined across all participants as incorporating standardized or simulated patients, manikin-based simulation, or both. Faculty experiences with any type of virtual simulation or laboratory exercises using role play were not included. Faculty who taught any physical therapy courses, regardless of curricular focus, for at least 1 year were included, as the emphasis was on overall faculty development experience with SBE rather than specific simulations. Faculty from the researcher’s own institution were excluded to allow for a larger range of participants.

### Data collection

**Fig. 1:**
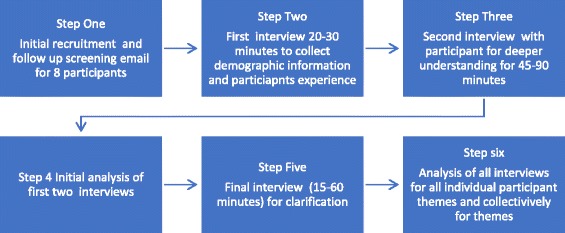
Data collection

A schedule was established that allowed multiple interviews to occur over 1 month’s time [19]. Interviews were semi-structured, involving six to ten open-ended questions created through the lens of transformative learning theory; each interview lasted no longer than 90 min [19]. The semi-structured interview questions were designed to uncover the participant’s story “sideways,” rather than formally in a top-down fashion [19]. Figure 1 illustrates the data collection process, and the semi-structured interview questions are found in Appendix 2.

To ensure that a common definition of SBE was used, participants completed an initial screening questionnaire. The initial interview gleaned demographic information and allowed the participants to describe their own definition of SBE. Participants described SBE as the use of standardized or simulated patients or computerized manikins. Virtual simulation or laboratory exercises using role play were not included as part of this study. Participants may have used those methods in their teaching, but the interviews focused on incorporating standardized or simulated patients and manikin-based simulation. The second interview sought to increase depth of understanding of each participant’s experience with SBE, with a focus on their personal narrative and reflection on their experiences [19]. The third and final interview was used to clarify the primary researcher’s understandings from the previous two interviews and also asked participants to review and reflect upon transcripts from their first two interviews. This three-interview approach, standard in IPA, promoted ongoing reflection and a deeper understanding of the participants’ experience [21]. In total, 24 semi-structured interviews (three with each participant) were conducted.

### Analysis

**Fig. 2:**
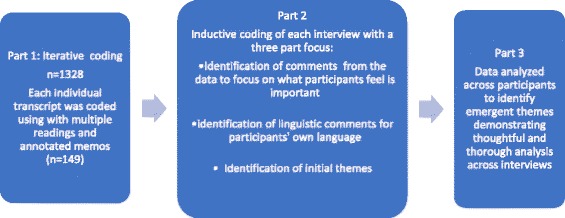
Analysis process

There is no single uniform method for data analysis using IPA. Smith et al. emphasized that IPA is not a prescription, but rather an examination of how the researcher makes sense of the data collected to provide a narrative story of the participants’ own sense-making [19]. The primary researcher spent significant time examining the data collected, reading and rereading each transcript to develop a general understanding of the individual participant’s story and the dialog that occurred between participant and researcher [19]. Data were coded during this initial review process using iterative and inductive codes that made sense of the participant’s own point of view [19]. After iterative and inductive coding, deeper coding was done with the aim of identifying descriptive comments that focused on what the participant felt was important. Descriptive comments were followed by linguistic comments that mirrored the language used by participants; the final step was thematic coding across participants [19]. Throughout the analysis process, analytic memos were used to record thoughts about the coding decisions made. After thoughtful and thorough analysis, the final themes were presented as findings. Figure 2 outlines the data analysis process.

Validity of individual participant stories was established through member checking, allowing participants to read, clarify, and verify their transcripts from the first two interviews. Thematic analysis was validated through expert review by the second author and two additional readers who reviewed the themes to determine whether they aligned with the extensive supporting quotes and the researcher’s field notes.

## Results

### Subjects

Eight physical therapist faculty simulation educators participated in this study. Each participant had been using SBE as part of his or her teaching practice from 3 to 19 years at the time of data collection. Participants were given pseudonyms to protect their anonymity in accordance with ethical review board. Table 1 provides an overall description of the individual participants, and Table 2 provides overall demographic information about the participant sample.

**Table 1:** Individual participant information

Participant	Years as a PT faculty member	Years teaching with simulation	Interprofessional simulation	Formal simulation training
Anne	13	6	Yes	Yes
Barbara	8	9	Yes	Yes
Connie	13	9	Yes	Yes
Donna	19	16	Yes	Yes
Erin	7	6	Yes	Yes
Felicity	11	11	Yes	No
Gordon	3	3	Yes	Yes
Henry	10	8	Yes	No

**Table 2:** Overall demographics as a percentage of participant sample

Demographic category	Number of participants
Male gender	2
Graduate program	8
Possess a terminal degree Ed.D., Sc.D., Ph.D.	4
Currently enrolled in a terminal degree program at time of study	3
Number of individual states represented in study	8
Participants who are licensed therapists in the USA	8
Participants that teach in program size over 50 students	5
Participants that teach in program size under 40 students	3

Six of the eight participants reported having received some formal SBE training. All participants were confirmed to have incorporated standardized or simulated patients and manikin-based SBE and used common language identified in the best practices from nursing [27].

### Individual participant profiles

Anne had included SBE in preparing students for clinical education for 6 years. Her entry into academia was not intended; in her words, “I wasn’t really planning on going into academia, but it was an open door.” Anne’s experience as an SBE educator and a clinical educator were interconnected. When meeting with students regarding clinical education experiences, she was “able to ask them more insightful questions” through the facilitation techniques she had learned through SBE. She believed that she was now interacting with students “in a more effective way.”

Barbara had taught with SBE for 9 years. She considered herself “a teacher now, but a physical therapist first.” Barbara’s experience was unique in that she “was involved as a clinician first with SBE with the university and making recommendations on what we thought we needed to go into SBE,” based on her clinical experiences as a physical therapist. She had been teaching with SBE longer than she has been a full-time faculty member.

Connie had been teaching with SBE for 9 years and embedded SBE in several courses throughout her curriculum. Connie’s opportunity to add SBE to her teaching practice began when the SBE program housed in another department asked for other disciplines to participate. She seized this opportunity to prepare her students for physical therapist practice.

Donna had been an SBE educator for 19 years and had the most experience among the participants. She led an interdisciplinary team of 35 SBE faculty members. Her initial experience with SBE was with standardized patients. Donna not only was almost entirely focused on interprofessional SBE but also had years of experience with physical therapist students in discipline-specific SBEs. As a certified healthcare simulation educator, she continued to refine her practice, looking “for new ways to measure the effectiveness of [SBE].”

Erin had been teaching with SBE for 6 years and began her SBE teaching after attending a formal SBE instructor course. Her experiences with teaching and learning with SBE led her to “get really frustrated that there’s really good evidence across health professions”, but her program did not fully “support” the use of SBE. She expressed a desire to expand the role of SBE in her teaching practice beyond her two courses, but because “I teach the classes by myself”, she was unable to do more.

Felicity had been teaching with SBE for 11 years, the same amount of time that she had been a faculty member. She described SBE as “a gift from God” and emphasized that the extensive resources and faculty staff support available through the center at her university helped her immensely with adding and continuing SBE as part of her teaching practice. Felicity did not undergo formal SBE training; she described herself as “a natural” indicating that she believed she had an innate ability to incorporate SBE without training.

Gordon was the newest SBE educator, having only 3 years of experience. His primary faculty role was clinical education. He reported SBE was “a job requirement” when he took his position. His training began with one-on-one mentoring from another physical therapist faculty member, as well as from nursing faculty. He attended courses and training that developed his SBE teaching practice. Gordon described the evolution of his experience teaching with SBE as simultaneous with his overall teaching career.

Henry had been teaching with SBE for 8 years and described his faculty development and training with SBE as “totally self-learned,” having not taken any coursework. He and two interprofessional colleagues at his university began their SBE practice by working together to investigate how to add SBE in their respective programs. Henry credited his program’s mission, which emphasizes the importance of SBE, and its extensive resources as important components of his start and evolution as a SBE educator. Henry said his path to adding SBE to his teaching practice was consistent with his overall personality, in that he learned by self-investigation.

### Common themes across participants

After understanding each individual participant’s perspective, qualitative analysis across participants revealed four themes related to faculty development and transformation. Each of the themes is presented below, along with supporting evidence made by at least one participant.

#### Theme 1: faculty development with SBE strengthens their professional identity as physical therapists

Participants were found to solidify their professional identity as physical therapists through SBE training. As they reflected on their professional work, they recognized how SBE training allowed them the opportunity to bring their previous experience into their work in a deliberate and conscious way. The specific experiences participants had as physical therapists were important influences on their creation of SBE scenarios. Faculty created cases based on something they experienced as a clinician that they thought was important for their students to learn. One distinct discussion point was the question of whether physical therapist students should participate in SBE experiences in which the manikin goes into cardiac arrest, a common issue in medicine and nursing. Some participants avoided this type of SBE:

*My patient [scenarios do not involve a cardiac arrest] because my goal isn’t to teach them how to respond to [cardiac arrest], I don’t think that’s necessarily an entry level skill that they must have upon entering their clinical experience.*

However, one participant’s clinical experience of having a patient go into cardiac arrest during her own physical therapy session was enough to convince her that all physical therapist students should experience it, even if other physical therapist SBE educators were “very critical of [her]”:

*I worked in acute care and remember, as a new grad, finding a patient [in cardiac arrest] and running from the room and not knowing what to do and feeling that sense incompetence in that area. I think my case grew out of that.*

The professional role of the physical therapist in the acute care setting, where almost all participants had worked, was described by participants as helping them with the flexibility needed to teach through SBE:

*It’s just like working with your patients, you have a plan and it never goes that way but, whatever ends up happening, it’s still a benefit to that patient.*

SBE helped participants isolate what was really needed in clinical practice. They found SBE to help identify “what the students really need to know, and trying to figure out what’s the best way for them to understand that.” As physical therapists, SBE allowed participants to be reminded to provide more “patient-centered care,” give “people choices,” and be “a better listener to patients.” Their experience as facilitators with SBE allowed them to take on the facilitator role with their patients. “I think it’s because, when you debrief you shut up, you listen, you get people talking. That’s been really good, understanding that there’s no right way.”

#### Theme 2: faculty development with SBE is affected by their introduction and training as SBE educators

Each participant’s SBE development was affected by their unique path. For many, the initial experience they had with using SBE in their teaching practice began as a need to learn a method of teaching:

*In my role as [Director of clinical education I] saw students struggling in acute care and because of my own background in cardio-pulm and in acute care, felt like it was such a unique environment that we weren’t really preparing our students for, and when I was introduced to simulation it was just like this light bulb that said this is how we can do this.*

The addition of simulation influenced their development through allowing them to reach their students through another method.

The presence or absence of training also influenced their development. Participants reported having some formal training and mentorship in SBE as an introduction to the SBE process. Participants took formal SBE courses before they started teaching with it. These courses were found to provide an overview of SBE and debriefing. Faculty attended courses alongside or after the start of their SBE teaching. Two participants did not attend any formal SBE training when they started, and reported primarily self-mentoring.

Participants discussed how they continued to seek development opportunities through conferences and through mentors.

*I’ve had training in SBE since 2007. I’ve been to different regional SBE conferences over my time as well as going to different presentations just kind of figuring out what other people are doing. So, some formal, some informal experiences as well.*

*[If] I don't have extra expertise or extensive experience in an area, then I'll consult with somebody who does to try to design the scenario to be as real as possible.*

Participants used terms such as “novice,” “natural,” “becoming an expert,” “advanced,” or “somewhat in the middle” when describing their own level of expertise with SBE. The participants with the greatest amount of overall experience discussed more creative uses of SBE than the other participants. Participants discussed trialing differing approaches to simulation, for example, transitioning to a delayed debriefing model with SBE to see if it was similar to the feedback they would see in the clinic. With this model, their students reflected for a longer time and then worked through debriefing later.

*We’re delaying [the debriefing] because when they become interns and when they get their job you don’t always have somebody who’s right there the second you’re done doing something where you can debrief with that person.*

#### Theme 3: the physical therapist faculty develop their interprofessional education through SBE

Participants embraced SBE for interprofessional scenarios. Interprofessional SBE scenarios either included students from other professions to participate alongside physical therapist students or incorporated confederates filled by faculty or actors to portray other professions such as nursing. Although experience with interprofessional SBE was not a requirement for study participation, each participant described their teaching practice as including interprofessional education. Participants were using SBE for interprofessional education at varying levels. Participants described interprofessional SBE as enhancing the fidelity of their scenarios, noting that because physical therapist do not routinely practice without interaction with other health care providers for patient care, incorporating other professions in the simulations mirrored what students would expect to find in the clinical environment. They reported that interprofessional SBE fostered “realistic” student engagement with other healthcare team members and enabled students to practice in a realistic team setting as part of an interprofessional team:

*[SBE] prepared [students] for some of the realities of healthcare in shaping the learning experiences that are going to get them ready to function in the interdisciplinary world, or interprofessional world that is, is healthcare.*

SBE was used to allow PT students to work among an interprofessional team and to understand each other’s roles:

*I try to have that come up in the debriefing about what more you do in your profession, what other kind of settings do you work in, [and] how does that differ if you’re at an outpatient site, versus an inpatient.*

Collaboration with other health professionals furthered the participants’ own teaching development and validated their use of SBE. Participants discussed collaboration during their SBE training, while running SBEs, and when mentoring other professionals. Experience with interprofessional faculty SBE training allowed them to describe the role of the physical therapist to other professions:

*I found that everybody was open to learning about what we did and it was an opportunity to explain to them what we did. They were surprised to know that we would take vital signs upon getting a patient out of bed. You know they looked at us like, you do that? And I said yes, we do.*

Interprofessional education was described as creating additional responsibility for the faculty members beyond educating physical therapists. Physical therapist faculty had to understand what the other professions’ role and scope of practice was. Knowledge of how each team member would interact and care for the patient in the scenario was required to create and execute interprofessional simulations. Working interprofessionally with faculty from other disciplines required attention to ensure that the learning objectives of an interprofessional experience met the needs of all learners.

*When I work closely with the instructor of the course or with our colleagues from occupational therapy, their instructors of the course, or with nursing, and really sit down with them and say, we really have deep conversations about what we’re trying to accomplish and looking to ensure that it fits course objectives.*

Interprofessional SBE extended beyond the classroom. There is an increase in physical therapist presence at interprofessional conferences over the past few years. The participants were no longer “in a room of physicians and nurses.” These interprofessional SBE experiences have “been huge” for understanding of other professions and led to a better appreciation of being a team player.

#### Theme 4: involvement with SBE facilitates professional growth of the physical therapist educator

Participants provided evidence that the experience of teaching with SBE had transformed their teaching and development. The more participants with SBE, the better they believed they were as SBE educators. The participants shared the various ways that teaching with SBE had transformed them.

For some, SBE made teaching more enjoyable and fulfilling. Participants reflected that it “keeps me really excited about what I do” and has “really been a positive experience.” Participants said that the more they taught through SBE, the more expertise they gained. This furthered their professional drive to be effective educators and develop their simulation education ability:

*There’s a comfort level in a simulation I’ve done before, I know exactly how the debrief is going to go, and I can facilitate that debrief in a more efficient and effective way*

*I’d much rather do SBE than anything else… It’s easier to probe and it’s easier to get people actively learning and involved, so I find that I can be more effective in a SBE.*

Participants discussed how their experiences with SBE informed their teaching in the classroom and changed them as an educator into the role of a facilitator. SBE worked to deepen faculty’s approach to student learning through questioning instead of lecturing. The faculty’s ability to educate in a deeper way had increased as they gained more experience.

*When I think about the first class I ever taught, I was lecturing the entire time and just downloading information. Now I spend most my time in the classroom working on synthesis and evaluation and integration of information, and I don’t get as concerned about they must know this knowledge.*

Participant credited SBE with changing their professional lives beyond the classroom.

Expertise with SBE led them to “gain a large amount of notoriety” on the national level, “receive awards,” and nominations increasing feelings of self-worth as an educator.

*I think one of the biggest light bulbs was not realizing until we had talked how much I had accomplished with everything….. I have achieved things that I’m really proud of and I try to convey to students you don’t have to be a straight A genius you need to have perseverance.*

Participants transformed their communication through their SBE experience.

*I’m a little more able to stop and slow down than I sometimes was before, because I’m a little, I think I’m a little bit better at reflecting in the moment.*

In addition to the above transformations, participants noted that they had advanced their practice through written or presented scholarship related to SBE, and all participants have worked on some aspect of SBE research.

## Discussion

The findings of this study provide an understanding that while each physical therapist faculty member’s development with SBE is individual, several experiences are common among them. The four themes identified in this study illustrate these commonalities and demonstrate key findings.

### Prior experience

The first theme speaks to how SBE influences a physical therapist faculty member’s identity. This finding is consistent with educational research in that an educator’s professional life experiences are known to influence his or her teaching philosophy and growth over time [29]. The common life experience for each participant was his or her clinical background as a physical therapist. The physical therapist knowledge and clinical mindset that was embedded in their teaching was found to predispose participants to certain assumptions and expectations. This aligns with cognitive learning theory, which describes how previous experience is essential to the learner’s process [30]. This embedded knowledge has been identified as part of the academic faculty process in other professions [31].

Participants drew on the mistakes they made to assist their students and were found to understand connections between what they had experienced and what they wanted their students to learn. Participants’ own learning as clinicians and how they were taught influenced their own teaching. This finding is common in higher education, where faculty have previous experiences as a student and from their professional craft outside the field of higher education [32]. Similar findings have been uncovered in physical therapist research. Hilliard examined physical therapists’ development of cultural humility [33]. In both this study and Hilliard’s, physical therapists were found to use previous life experiences and focus on the patient to place their experiences in meaningful context.

### Training

In this study, training varied among participants. Some faculty received training prior to or during the start of their simulation teaching, while two reported not having formal training. Regardless of how they were trained, faculty are responsible for the best practices in debriefing, which include intentional preparation and an established plan for accomplishing the stated objectives of the simulation [34]. Faculty may attend formal training programs, degree programs, or fellowships to acquire these skills [14]. Training-the-trainer programs were used by many faculty, who relied on mentors or peers to self-investigate teaching methodology in addition to or instead of formal training. The literature states that training the trainer requires a well-developed curriculum if it is to be successful [35]. Despite existing literature on the importance of formal training, this study provides evidence that it is possible for a physical therapist educator to function in the role of a simulation educator without formal training. However, while their self-reported experiences align with guidelines from the literature and the other participants’ experiences with formal training, we did not observe them and cannot draw conclusions about how effective they fulfill the role.

Following the best practice standards in many professions, the faculty member must understand the tenets of simulation, from basic terminology to scenario creation to facilitated debriefing [15]. While participants discussed consistent elements with these best practice standards, not all participants sought formal training. This illustrates either a lack of understanding of the benefits of formal training in the literature or a lack of recommended standards for SBE within the profession of physical therapy in the USA. Further conclusions on how training influenced participants’ SBE and the overall effectiveness of their teaching cannot be drawn because faculty were not directly observed in this study. It is not clear if the faculty who did not attend training may have been more successful if they had, or if those who had training are more successful, only that participants were incorporating SBE in physical therapist education based on a variety of training methods.

### Interprofessional education

A consistent theme across participants was the interconnectedness of SBE and interprofessional education. Participants relied on SBE for teaching interprofessional content such as teamwork and communication. Simulation education is recognized in nursing best practice as an effective process to develop learners’ interprofessional teamwork skills [36]. Specific to physical therapy, Bagatell and Broggi found that having physical therapy and occupational therapy students and faculty work together through interprofessional SBE helped address role misconceptions, demonstrate the importance of communication, and increase confidence [37]. Buckley et al. concluded that students from a variety of professions (medicine, nursing, and other health professions, including physical therapy) who participated in an interprofessional SBE experience increased their perception of the importance of interprofessional teamwork [38].

The experiences the participants had as physical therapist educators teaching within interprofessional simulation education required a commitment to interprofessional collaboration with other health profession faculty. Participant responses suggested that they were part of, or were working toward being a part of, an interprofessional simulation community of practice. Participant experiences with this are mirrored in the literature. Hargreaves and Fink emphasized that sustained communities require a commitment to the task and the community [39]. This commitment includes the ability to evolve with mutual respect and understanding [39]. Participant experiences working with other faculty were seen to align with literature on cultural relationships in higher education. Participants discussed strategies they used to work as a team, much like Bui and Baruch, who asserted that an understanding of culture and how different professional cultures work together is essential in higher education. Participants also had experiences working with students to examine what limited their understanding of the roles of other professions [40]. This meshes with Frederick et al., who demonstrated that their perceptions and biases about their own profession are apparent and acknowledged within a student simulation [26].

Interprofessional education’s intentions of enhancing fidelity through mirroring the physical therapists’ clinical environment and fostering teamwork through shared learning objectives are both consistent with the literature’s recommendations on successful interprofessional simulation [41]. This study did not seek to examine interprofessional education beyond the experiences participants reported, so further conclusions about how interprofessional SBE influenced faculty members’ teaching practice cannot be drawn.

### Professional transformation

Cranton asserted that teachers learn to teach through the practice of teaching itself and that teaching transforms more than teachers’ educational practice [42]. Each participant reported they transformed through their teaching practice with SBE. Participants were asked to reflect on their teaching practice through this research study. For some participants, transformations involved professional development and enjoyment of teaching. An educator’s life experiences are known to influence their teaching philosophy and development over time [29]. In examining their own transformations, participants reported deliberate practice, in that SBE was an initial dilemma for them and they needed ongoing experience and practice in order to improve. While one participant might be a self-described “natural,” others relied on ongoing practice to learn the skill. These reports of working to improve the educational practice highlight that their focus on developing SBE was deliberate [43]. Participants transitioned from merely using a teaching method into its becoming part of their teaching purpose; the learning they received from this teaching carried over to their professional lives.

Mezirow discussed how critical reflection is an essential component of transformation [22]. Transformational learning occurs when people encounter a dilemma requiring action and self-reflection, a situation that accounts for the learners’ awareness and reflection upon their own emotional response within their learning [44]. For others, the way they communicated and related to others including patients was transformed. Simulation education is seen to transform the careers of educators through opportunities, such as notoriety and awards, and areas to pursue scholarship. The use of the IPA methodology in this study provided space for this ongoing reflection because the researcher fostered reflective conversations through a multi-interview approach [19].

### Implications for the physical therapist faculty member

This study illustrates the physical therapist faculty development process when incorporating SBE in a way that has not previously been reported. It provides a valuable understanding of what physical therapist educators experience when they teach with SBE. The participant descriptions of including SBE in their teaching were consistent with other documented professions. However, physical therapist educators may be limited by the absence of guidelines or frameworks in other professions that emphasize standardized training, best practice SBE for the profession [45, 1]. In addition, the participants and the researcher had a limited awareness of simulation beyond the context of physical therapist education in the USA outlining a need for increased global collaboration. Physical therapist faculty looking to adopt SBE teaching can use this study to begin to understand the experience of an SBE physical therapist educator and to make informed decisions for their own professional development. New educators can expect to rely on their clinical experiences as they begin and require ongoing deliberate practice, often informed by training, to develop their expertise with SBE. Lessons learned from this study promote an understanding of the best practices in SBE and interprofessional education that are beneficial to physical therapist SBE.

This study recommends a greater emphasis on relying on best practice documents from other professions and the need to establish the profession’s own best practices for SBE. The wealth of SBE research in other medical and non-medical field medicine should be examined in light of physical therapy teaching practice and development.

The profession of physical therapist education should seek to understand and define what is expected with training requirements for SBE teaching. Attention to this matter from those with experience with simulation within the profession is warranted. Other professions have defined training as a prerequisite for success [46, 35]; however, this study provides evidence that faculty in physical therapist education are teaching without training. Participants in this study reported they execute SBE practice in a manner consistent with other professions that have varied levels of training. While faculty members looking to add SBE to their teaching practice should not let the absence of training deter them from starting, they should at minimum seek mentors or self-training modules to inform their practice as they work to attend more formal training programs.

This study provides evidence that understanding faculty development resulting from the use of emerging teaching methods can provide a wealth of information that may be of benefit if repeated in other disciplines. Each profession that defends, defines, and describes the use of SBE adds to the collective body of knowledge on its use and importance in education. The faculty perspective was emphasized in this study, which allows for a view not otherwise known in the specific profession studied. Other health professions should seek to examine the faculty experience with SBE through valid research methods.

### Limitations

There were several limitations to the study. The study used only one format for data collection, interviews, rather than a mix of data collection methods that included direct observation. Direct observation may have led to differing findings and allowed triangulation to strengthen them. The small number of participants also did not make the findings generalizable.

However, the personal account of each participant provided unique information. This study was designed to describe particular individual experiences and compare them for commonalities. It purposely excluded a more general understanding of the phenomenon of SBE in physical therapy.

The methodology chosen may be viewed as a limitation due to its emphasis on subjectivity. IPA seeks to understand a participant’s subjective interpretation of what is happening during a specific life circumstance through a shared interpretation between the participant and the researcher [18]. This study followed a detailed approach thoroughly and thoughtfully to create results worthy of scholarly acceptance [19]. The results of this study are transferable, but not generalizable. While the eight participants share commonalities, they do not represent all physical therapist SBE faculty educators. The study only included participants from accredited physical therapist programs in the USA in order to align teaching standards and curriculum. While participants from other countries were excluded, literature about global SBE practices was included. However, limits to common research and lack of rigor in physical therapist education studies limited these conclusions.

## Conclusion

This study sought to understand individual physical therapist faculty development with SBE and identify commonalities across eight participants. Four themes highlighted SBE enhanced physical therapist faculty member’s professional identify, was informed by introduction and training, was inter-connected with interprofessional education, and led to professional transformation. In addition to what is known from literature in other professions, knowledge gained in this study provides insight for physical therapist faculty who want to understand what they may experience when incorporating SBE in their teaching practice. Ongoing research and collaboration to develop and expand the teaching practice of SBE as part of physical therapist education is warranted.

## Appendix 1

### Screening email

Hello. Thank you for your interest in participating in this study examining faculty’s experience with simulation education. This screening email is being sent to you because you indicated you are interested in being a participant for this study. Please answer the four brief questions on information needed to ensure findings from this study would represent rigorous doctoral work. I appreciate your time in answering the following brief questions.

Questions:

1. How long have you been teaching with simulation education?
2. Which courses or physical therapy content areas do you utilize simulation education?
3. What preparation and training have you had in simulation?
4. What is your definition of simulation education?
  Thank you. I appreciate your time. Participants in the study need to meet the criteria of a minimum amount of time using simulation and a shared definition. If you meet these inclusion criteria, you will be notified via email within the next seven business days to be part of this study and further information will be sent at that time including consent to participate.

## Appendix 2

### Interview Questions

Interview questions that addressed the research question: How do physical therapist faculty develop while including simulation based education and are there commonalities between educators?

O Overall demographics

- Years as a faculty member?
- Clinical practice area?
- Rank?
- Area of the country?
- Freshman entry or graduate entry program?
- Presence of simulation center at their institution?
- Level of simulation training (defined as certified instructor, attended a comprehensive instructor workshop, attended informal instructor trainings through APTA or other program, or on the job training)?
- Level of faculty education, terminal degree, board specialization?

Q2: What is your background as a physical therapist and an educator?

Prompts: Ask about clinical practice, how long in academia and courses taught.

Q Q3: Can you describe the type of physical therapist program you teach in?

Prompts if needed: How large? Type of student?

Q Q4: Can you tell me about your decision to start adding SBE to your teaching practice?

Prompts if needed: How did you decide to start? Which courses? Who or what motivated you?

Q Q5: I would like to hear about your experiences with training and orienting to SBE?

Prompts if needed: How did you get started? How did you learn how to do it? Any formal training?

Interview 2:

Q1: When we spoke last time, we talked about how you got started with SBE in your teaching practice. Now we are going to discuss more in depth your teaching experiences with SBE. First I would like to talk about the process you go through designing a simulation for your students, how do you get started? Is there a process or similar experience you go through?

Q2: Can you give me a few examples of SBE you have designed?

Q3: Can you talk about how you decide what is important to include with the experience?

Q4: Now let’s move on to discussing the execution of the actual SBE itself. What is that experience like for you? Can you share with me a few experiences? (Cues here will be from previous interview. Such as “for example you said you use it in your geriatrics course….”)

Q5: How would you describe your experiences within an SBE that did not go well?

Q6: How would you describe a successful SBE outcome for you?

Q7: Now that we have discussed how you go started and your experiences in your teaching practice, I’d like to talk about whether you think you have changed how you include SBE since you started teaching with SBE?

Q8: In your experience has SBE impacted your students’ learning?

Q9: Have you learned anything about yourself through teaching with SBE?

Q10 Aside from adding SBE to your practice as a method, has teaching with SBE changed you as an educator?

Interview 3:

No pre-created questions were used. This was a final interview for clarifying information from the previous interviews and for capturing participants’ reflective responses on their previous two interviews after reviewing their transcripts.
